# Early Rearing Conditions Affect Monoamine Metabolite Levels During Baseline and Periods of Social Separation Stress: A Non-human Primate Model (*Macaca mulatta*)

**DOI:** 10.3389/fnhum.2021.624676

**Published:** 2021-04-09

**Authors:** Elizabeth K. Wood, Natalia Gabrielle, Jacob Hunter, Andrea N. Skowbo, Melanie L. Schwandt, Stephen G. Lindell, Christina S. Barr, Stephen J. Suomi, J. Dee Higley

**Affiliations:** ^1^Department of Psychology, Brigham Young University, Provo, UT, United States; ^2^Department of Neuroscience, Brigham Young University, Provo, UT, United States; ^3^Laboratory of Clinical Studies, National Institute on Alcohol Abuse and Alcoholism, Bethesda, MD, United States; ^4^Section of Comparative Behavioral Genomics, National Institute on Alcohol Abuse and Alcoholism, National Institutes of Health, Rockville, MD, United States; ^5^Section of Comparative Ethology, Eunice Shriver Kennedy National Institute of Child Health and Human Development, National Institutes of Health, Poolesville, MD, United States

**Keywords:** dopamine, early rearing, monoamine metabolites, norepinephrine, rhesus macaque, serotonin, serotonin transporter genotype, social separation

## Abstract

A variety of studies show that parental absence early in life leads to deleterious effects on the developing CNS. This is thought to be largely because evolutionary-dependent stimuli are necessary for the appropriate postnatal development of the young brain, an effect sometimes termed the “experience-expectant brain,” with parents providing the necessary input for normative synaptic connections to develop and appropriate neuronal survival to occur. Principal among CNS systems affected by parental input are the monoamine systems. In the present study, *N* = 434 rhesus monkeys (233 males, 201 females) were reared in one of two conditions: as mother-reared controls (MR; *n* = 269) or without adults with 24-h access to same-aged peers (PR; *n* = 165). When subjects were six-months-old, they underwent a separation paradigm involving 4, sequential, four-day social separations from their mothers or peers, with each separation followed by three-day reunions with their mothers or their peers. Prior to the separation paradigm, baseline cisternal CSF samples were obtained, as well as at the end of each the four social separations, and after final separation, during a recovery period. CSF was assayed for concentrations of monoamine metabolites and a blood sample was genotyped for the serotonin transporter (5-HTT) genotype. Replicating earlier landmark findings, PR subjects with the *s* allele exhibited lower baseline concentrations of the serotonin metabolite 5-hydroxyindoleacetic acid (5-HIAA), when compared to PR subjects homozygous for the *L* allele. MR subjects were undifferentiated by genotype. PR subjects exhibited lower CSF 5-HIAA concentrations during baseline, but higher CSF 5-HIAA during social separations, when compared to MR subjects. There were rearing effects for the dopamine metabolite homovanillic acid (HVA) and for the norepinephrine metabolite 3-methoxy-4-hydroxyphenylglycol (MHPG), with PR subjects showing higher HVA and lower MHPG when compared to MR subjects. These findings indicate that there are long-term deficits in the response of monoamines following early maternal absence. The results of this study confirm and extend earlier findings that early parental absence has deleterious consequences for the development of the monoamine systems, and that these consequences are modulated by the 5-HTT genotype.

## Introduction

In primates, most cortical brain development occurs postnatally (Bakken et al., 2016). Shortly before and immediately following birth, infants experience an explosive process of synapse formation, known as exuberant synaptogenesis (Eayrs and Goodhead, 1959; Altman, 1972; Wang et al., 2009; Fox et al., 2010; Naskar et al., 2019). This period is followed by systematic pruning of the overabundant connections, along with synaptic strengthening in functional neuronal pathways, with both potentiated by experience (Huttenlocher and Dabholkar, 1997; Hashimoto and Kano, 2003; Fox et al., 2010). Studies show that mothers play a critical role in this process of synaptic maintenance and pruning, a process necessary for normative brain development (Sánchez et al., 1998; Scher et al., 2009; Fox et al., 2010; Pollak et al., 2010; Nelson et al., 2011; Schneider et al., 2012). Developmental studies suggest that parental sensitivity is necessary for normative neurobiological development to occur, providing the right input at the right time, something Greenough and colleagues referred to as the “experience-expectant brain” (1987).

Without the appropriate social and physical stimuli that are typically provided by a sensitive caregiver, the “experience-expectant brain” shows marked deficits. For example, studies of humans raised in institutions exhibit fewer synaptic connections relative to their typically-reared peers, resulting in smaller brains (Dobrova-Krol et al., 2008), reduced white matter (Eluvathingal et al., 2006; Mehta et al., 2009), reduced functional connections between structures in the limbic system (Chugani et al., 2001; Daniels et al., 2013; Kumar et al., 2014), increased amygdalae volume (Rutter et al., 2007; Mehta et al., 2009; Tottenham et al., 2010), and decreased corpus callosum volume (Mehta et al., 2009). They also tend to show a dysregulated stress response (Gunnar et al., 2001; Tarullo and Gunnar, 2006). While Nelson et al. (2011) makes the case that it is difficult to tease out the deficits resulting from impoverished early environments and the deficits resulting from poor social experiences, Studies of children with less severe early experiences, who develop in homes with more stimulating surroundings but whose mothers are less responsive to their infants’ needs, for instance, mothers who suffer from depression, also show deficits in CNS development and functioning (Ashman and Dawson, 2002; Pruessner et al., 2004; Qiu et al., 2015); albeit, not as extreme as those seen in institutionally-reared children.

While it is difficult to measure neurotransmission and monoamine functioning in children, studies suggest that individuals who were abused in childhood demonstrate higher concentrations of dopamine than controls (de Bellis et al., 1994, 1999; Egerton et al., 2016), as do individuals reporting low maternal care (Pruessner et al., 2004) or neglect (Roy, 2002). Childhood abuse is also associated with low serotonin transporter binding (Miller et al., 2009) and studies suggest that early life neglect and abuse are linked with impaired serotonin functioning (Roy, 2002; Miller et al., 2009). Maltreatment is also associated with elevated norepinephrine concentrations (de Bellis et al., 1999; Otte et al., 2005). Some studies have assessed the role of the early environment on neurotransmission in the context of the serotonin transporter gene (5-HTT), which has two major variants: a more frequently occurring long allele (*L*), and a more recent, less frequently occurring short allele (*s*). The *s* allele of 5-HTT genotype is associated with low transcriptional efficiency, resulting in reduced serotonin expression (Lesch et al., 1996; Little et al., 1998). In humans, studies show that the *L* allele of the 5-HTT genotype confers resilience to maltreatment (Caspi et al., 2010) and that it has a protective effect against the development of insecure attachment in children who were institutionalized as infants (Bakermans-Kranenburg et al., 2012; Humphreys et al., 2015). However, such early rearing aberrations lack experimental control and are often intertwined with parental-child genetic risk.

Due to the difficulties experimentally studying the role of early experience on the brain in human subjects, translational animal models are frequently used to study the impact of parents on infant development. In these models, early rearing conditions can be experimentally-randomized and the environment can be controlled. There is an extensive history of using rhesus monkeys (*Macaca mulatta*) to model the importance of early maternal care on normative development (Harlow, 1958; Suomi, 1995; Kuhn and Schanberg, 1998). Rhesus monkeys are ideally-suited for these types of experiments as they show a high degree of genetic similarity to humans (Gibbs et al., 2007), which is particularly important because, like humans, rhesus monkeys possess an analogous biallelic 5-HTT genotype (Bennett et al., 2002). As in humans, the mother-infant attachment relationship is critical for normative development to occur (Harlow, 1958, 1969); indeed, attachment theory was based, at least in part, on Harlow’s studies of rhesus monkeys (van der Horst et al., 2008; van Rosmalen et al., 2020). Rhesus monkeys also show parallel developmental periods (Machado and Bachevalier, 2003; Suomi, 2005), although at an accelerated rate (Roth et al., 2004), allowing researchers to assess developmental outcomes in a relatively rapid time-frame.

To study the effects of caregivers on infant outcomes, the rearing paradigm most commonly used in rhesus monkeys involves randomly assigning infants to one of two conditions during the first 6 months of life. The experimental group includes peer-reared subjects (PR), a condition where infants are removed from their mothers at birth, hand-reared in a neonatal nursery for 30 days, and then housed together in groups of 2–4 without adults. The controls are reared by their mothers [mother-reared (MR)] in social groups comprising of same-aged infant peers, their mothers, and two adult males, a social condition that approximates the natural rhesus social setting. While PR monkeys are provided ample opportunity for social interactions and live in physical environments that parallel MR subjects, research shows that PR monkeys exhibit altered neurochemical measures (Higley et al., 1991b, 1996, 1992; Clarke et al., 1996; Shannon et al., 2005) and neuroanatomical outcomes (Sánchez et al., 1998; Spinelli et al., 2009, 2010), although the sample sizes for these studies are relatively small, and the majority of these studies have generally not assessed the impact of early maternal absence on neurotransmission as it relates to chronic stress and even less often during a recovery period following the stressors.

The central monoamine system of the rhesus monkey parallels humans in many ways, showing similar resting activity and increased turnover in response to acute and chronic stress (Higley et al., 1991b, 1992, 1996; Clarke et al., 1996; Higley and Bennett, 1999; Shannon et al., 2005). Studies show that serotonin is lower in PR subjects both at baseline (Clarke et al., 1996; Shannon et al., 2005; Spinelli et al., 2010) and during acute stress (Clarke et al., 1996; Bennett et al., 2002), when compared to MR subjects. However, studies have not assessed differences between MR and PR monkeys during conditions of chronic stress nor during post-separation recovery.

Some individuals tend to be relatively resilient to the effects of such early rearing deficits. Caspi and Moffit’s landmark study in humans (2002), as well as work published at the same time in rhesus monkeys (Bennett et al., 2002), explain such resilience as resulting from gene X environment interactions. To illustrate, Bennett et al. (2002) showed that PR subjects exhibited reduced CNS serotonin activity, as measured by low cisternal CSF concentrations of the serotonin metabolite 5-hydroxyindoleacetic acid (5-HIAA). However, when the PR subjects were categorized by serotonin transporter genotype, PR subjects homozygous for *L* allele were relatively unaffected by maternal absence, showing CSF 5-HIAA concentrations that were similar to MR subjects. On the other hand, PR subjects with the *s* allele showed low CSF 5-HIAA concentrations, while MR subjects’ CSF 5-HIAA concentrations were undifferentiated by genotype. Such studies suggest that genetic variation may convey resilience to individuals with deleterious early rearing experiences. The present study seeks to replicate this prior finding in an independent sample.

In the present study, the effect of rearing on monoamine metabolite functioning is assessed during baseline, following a repeated social separation stressor, and during post-separation recovery in a large sample of infant rhesus monkeys. Then in an effort to replicate earlier findings (Bennett et al., 2002), subjects are stratified by 5-HTT genotype and their CSF 5-HIAA concentrations are assessed. It was hypothesized that early maternal absence would be associated with altered monoamine measures prior to, during, and following a well-characterized social separation paradigm, and that the 5-HTT genotype would influence stress-induced changes in CSF 5-HIAA.

## Materials and Methods

Subjects were *N* = 434 infant rhesus monkeys (233 males, 201 females), housed at the National Institutes of Health Animal Center in Poolesville, MD, United States. All subjects were randomly assigned to one of two rearing conditions at birth as part of a larger research program: mother-reared controls (mothers; *n* = 269) or reared with same-aged peers in the absence of adults (*n* = 165). See Shannon et al. (1998) for a detailed description of rearing conditions. Briefly, MR subjects were in born into and reared in social groups comprised of eight-to-twelve adult females, two adult males, and one-to-two other same-aged infants for the first 6 months of life. This condition approximates the social composition of the wild rhesus monkey, albeit smaller in size, than a typical rhesus monkey troop (Lindburg, 1971). PR subjects were separated from their mothers immediately following birth and hand-reared in a neonatal nursery for the first 30 days of life. Following the first 30 days, PR subjects were housed with constant access to three other similarly-reared age-mates. The physical housing for both MR and PR conditions consisted of indoor-outdoor enclosures (indoor: 2.44 × 3.05 × 2.21 m; outdoor: 2.44 × 3.0 × 2.44 m), with a 12-h (0700–1900) light-dark cycle for the indoor enclosure and natural seasonal light cycles for the outdoor enclosure.

To assure that any rearing effects were not the result of dietary differences, based on an earlier study from our nursery showing the importance of essential amino acids on CNS development and behavior (Champoux et al., 2002), the PR infants were hand-fed a 50:50 mixture of Similac (Ross Laboratories, Columbus, OH, United States) and Primalac (Bio-Serv, Frenchtown, NJ, United States) formula supplemented with physiologically relevant concentrations of DHA (1.0%) and arachidonic acid (1.0%) every 2 h until they reached 30 days of age. From day 30 until 4 months of age, formula was administered at 400 mL/day, and standard high-protein monkey chow was gradually introduced to the diet, and the infants were provided *ad libitum* access to water. At 4 months, the amount of formula provided was reduced to 300 mL/day, and, at 5 months, it was reduced to 200 mL/day. Following the nursery experience, both groups were fed commercial monkey biscuits and fruit once daily, and water was available *ad libitum*.

All protocols and procedures employed in this study were ethically reviewed and approved by the NIH Animal Care and Use Committee before beginning this study. This study was conducted in compliance with the National Research Council’s Guide for the Care and Use of Laboratory Animals, the United States Public Health Service’s Policy on Humane Care and Use of Laboratory Animals, and the Guide for the Care and Use of Laboratory Animals.

### Separation Paradigm

When subjects reached 6 months of age, they were captured and separated from their mothers or their same-aged peers for four sequential, 4-day-long social separations, each followed by 3 days of reunion with their mothers or with their peers. Separation procedures are described in detail elsewhere [see Spinelli et al. (2007)]. Briefly, the social separation paradigm began on Monday afternoon at 1,300 h, with the mothers or the same-aged peers’ removal from their respective social group. The subject remained in their homecage with the rest of the social group. On day 5 (Friday), the mother or peers were reunited with the infant in the homecage and a three-day reunion period ensued. This procedure was repeated weekly for a period of 4 weeks.

### CSF Sampling

**FIGURE 1:**
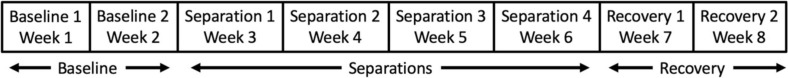
Study timeline. Depicts the timeline for the study, including when all samples were obtained. Baseline 1 included 312 subjects sampled for CSF 5-HIAA concentrations, 258 subjects sampled for CSF MHPG concentrations, and 298 subjects sampled for CSF HVA concentrations. Baseline 2 included 260 subjects sampled for CSF 5-HIAA concentrations, 198 subjects sampled for CSF MHPG concentrations, and 232 subjects sampled for CSF HVA concentrations. Separation 1 included 293 subjects sampled for CSF 5-HIAA concentrations, 260 subjects sampled for CSF MHPG concentrations, and 302 subjects sampled for CSF HVA concentrations. Separation 2 included 239 subjects sampled for CSF 5-HIAA concentrations, 195 subjects sampled for CSF MHPG concentrations, and 236 subjects sampled for CSF HVA concentrations. Separation 3 included 220 subjects sampled for CSF 5-HIAA concentrations, 176 subjects sampled for CSF MHPG concentrations, and 222 subjects sampled for CSF HVA concentrations. Separation 4 included 289 subjects sampled for CSF 5-HIAA concentrations, 244 subjects sampled for CSF MHPG concentrations, and 286 subjects sampled for CSF HVA concentrations. Recovery 1 included 258 subjects sampled for CSF 5-HIAA concentrations, 222 subjects sampled for CSF MHPG concentrations, and 244 subjects sampled for CSF HVA concentrations. Recovery 2 included 213 subjects sampled for CSF 5-HIAA concentrations, 182 subjects sampled for CSF MHPG concentrations, and 215 subjects sampled for CSF HVA concentrations. All samples were obtained on Thursdays at 1300 hours.

Baseline, separation, and recovery concentrations of CSF were sampled from subjects on eight occasions: *Baseline*—one and 2 weeks prior to the separation paradigm; *Separation Stressor*—on the last day of each of the four social separations (Thursdays, prior to reunion); *Recovery*, one and 2 weeks following the final social separation. See Figure 1 for a summary of the study timeline. On each occasion, CSF samples (1 mL) were collected from the cisterna magna by needle (22-gauge) and syringe (5-cc) *via* spinal puncture, under ketamine hydrochloride anesthesia (15 mg/kg, IM). To avoid the potential for a movement injury, the unconscious subjects were restrained in a lateral recumbent position, holding the head and hips to assure no movement during CSF removal. Ketamine was administered within 10 min and CSF was removed within 30 min of initial disturbance. Studies indicate that when samples are obtained within 30 min of entering the subjects living quarters, acute ketamine administration has no measurable effect on cisternal CSF monoamine metabolite concentrations in rhesus monkeys (Brammer et al., 1987). Samples were flash frozen in dry ice and stored at −70°C until quantified, as previously described [See Agartz et al. (2003) for additional details].

In brief, samples were thawed and 200-μl aliquots and 20-μl of the internal standard, F-HVA (650 pmol), were placed in 10,000 mW cut-off filters (Amicon, Beverly, MA, United States), which were pre-rinsed with water to remove the glycerol, and centrifuged at 10,600 × *g* for 20 min at 4°C. The column used was a Microsorb Short-One, C-18 (Rainin, Woburn, MA, United States) with a Guard-Pak precolumn containing Nova-Pak, C-18 (Waters, Milford, MA, United States). The buffer was 0.05-M sodium acetate, 0.021-M citrate, and 0.25-mM EDTA, pH 4.4. The organic was 5.5% (v/v) acetonitrile and 2% (v/v) methanol. The flow rate was 0.6 ml/min. Two liters of mobile phase was recirculated. Column temperature was 30°C. Standards of MHPG-hemipiperazinium salt, HVA and 5-HIAA were purchased from Sigma (St. Louis, MO, United States). F-HVA was a gift from Kenneth Kirk (NIDDK, Bethesda, MA, United States). Samples with gross blood contaminations (>2%, as indicated by pink coloration) were excluded. Inter- and intra-assay coefficients of variation were less than 10%.

### Blood Sampling and Genotyping

Blood (2 mL) was sampled from subjects using a vacutainer (EDTA) and needle (22-gauge) *via* femoral venipuncture under ketamine hydrochloride anesthesia (15 mg/kg, IM), within 10 min of initial disturbance. Blood samples were genotyped for the 5-HTT genotype using procedures described elsewhere (Barr et al., 2004). The serotonin transporter gene promoter region (rh-5HTTLPR) was amplified from 25 ng of genomic DNA with primers (stpr5, 5′-GGCGTTGCCGCTCTGAATGC; intl, 5′-CAGGGGAGATCCTGGGAGGG) in 15-μL reactions with Platinum^TM^ Taq and the PCR_*X*_ Enhancer System kit, according to the manufacturer’s protocol (Invitrogen^TM^, Carlsbad, CA, United States). Amplifications were performed on a Perkin Elmer^®^, (Wellesley, MA, United States) thermocycler (9700) with one cycle at 96°C for 5 min, followed by 30 cycles of 94°C for 15 s, 60°C for 15 s, 72°C for 30 s, and a final 3-min extension at 72°C. Amplicons were separated by electrophoresis on a 10% polyacrylamide gel, and the short (*s*, 388 bp) and long (*L*, 419 bp) alleles of the rh5-HTTLPR were identified by direct visualization after ethidium bromide staining. The accuracy of the PCR was further confirmed by cutting the bands, eluting and performing Sanger sequencing. See Supplementary Figure 1 for a representative gel and Table 1 for sample size distributions.

**TABLE 1:** Distribution of 5-HTT genotypes across rearing groups.

	***LL***	***Ls/ss***
MR	133	93
PR	67	27

*Includes the sample size and 5-HTT genotype distribution for each of the rearing groups included in the study.*

### Data Analysis

Mixed design, repeated measures analyses of variance (ANOVAs) were utilized to assess the relationship between rearing condition and social separation on CSF monoamine metabolite concentrations.

As they were significantly and positively correlated, the mean of the two *Baseline* samples and the mean of the two *Recovery* samples for the CSF monoamine metabolite concentrations were calculated and used in all analyses. The repeated measure independent variable was *Baseline*, *Separations 1, 2, 3*, and *4*, and *Recovery*. Rearing (MR or PR) was the between-groups independent variable. Unlike an earlier study on a small number of subjects (Higley et al., 1992), there were no sex differences for any of the monoamine metabolite concentrations. A two-way ANOVA was utilized to assess the relationship between 5-HTT genotype and rearing condition on the CSF 5-HIAA concentrations, with rearing condition and 5-HTT genotype as independent variables and CSF 5-HIAA concentrations as the dependent variable. CSF 5-HIAA concentrations were standardized within cohort year, as was done previously [see Bennett et al. (2002)]. All data were analyzed in SPSS, version 26.

## Results

### Serotonin Metabolite (5-HIAA)

**FIGURE 2:**
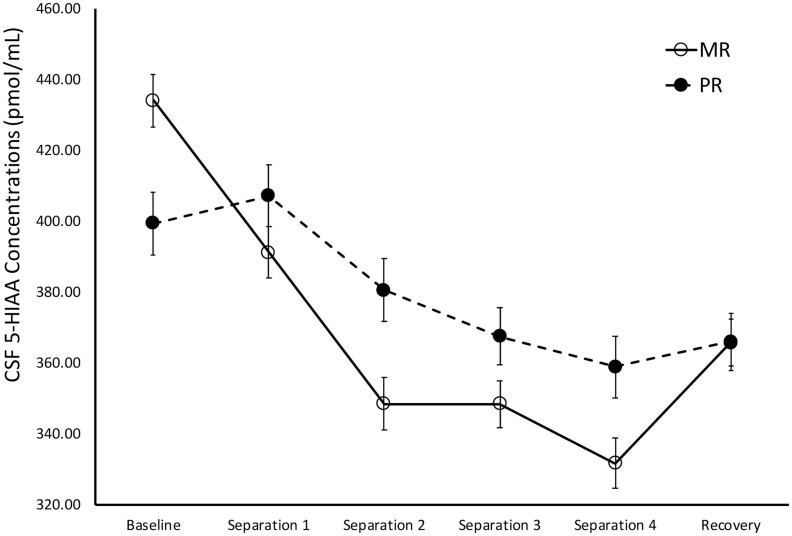
Rearing effects on baseline and stress-induced CSF 5-HIAA concentrations. Depicts the relationship between rearing condition and CSF 5-HIAA concentrations during *Baseline, Separations 1–4*, and *Recovery*. There was a significant main effect of time on CSF 5-HIAA concentrations [*F*(1,173) = 111.43, *p* < 0.0001], with CSF 5-HIAA concentrations higher during *Baseline* and *Separation 1*, when compared to *Separations 2–4* or *Recovery*. There was also a significant two-way separation-by-rearing condition interaction on CSF 5-HIAA concentrations [*F*(1,176) = 6.05, *p* = 0.02], with MR subjects exhibiting higher CSF 5-HIAA concentrations during *Baseline*, but lower CSF 5-HIAA concentrations during *Separations 1–4*, when compared to PR subjects. MR subjects are represented by white circles and solid lines, PR subjects are represented by black circles and dashed lines.

There was a significant main effect of time on cisternal CSF 5-HIAA concentrations [*F*(1,173) = 111.43, *p* < 0.0001]. Further analyses showed that CSF 5-HIAA concentrations obtained during *Baseline* and *Separation 1* were significantly higher than *Separations 2–4* or *Recovery* (*p* < 0.0001). There was also a significant two-way separation-by-rearing condition interaction on CSF 5-HIAA concentrations [*F*(1,173) = 5.97, *p* = 0.02], with MR subjects exhibiting higher CSF 5-HIAA concentrations during *Baseline*, when compared to PR subjects (*p* = 0.007). This effect reversed during the stress of the social separations, with MR subjects showing lower CSF 5-HIAA concentrations during *Separation 1 (p* = 0.04), *Separation 2* (*p* = 0.003), *Separation 3* (*p* = 0.04), and *Separation 4* (*p* = 0.007), when compared to the PR subjects. PR subjects showed an increase in CSF 5-HIAA concentrations, but PR and MR subjects showed no difference from *Separation 4* to *Recovery* (*p* = 0.60), and neither group returned to *Baseline* levels during *Recovery*. See Figure 2.

### Norepinephrine Metabolite (MHPG)

**FIGURE 3:**
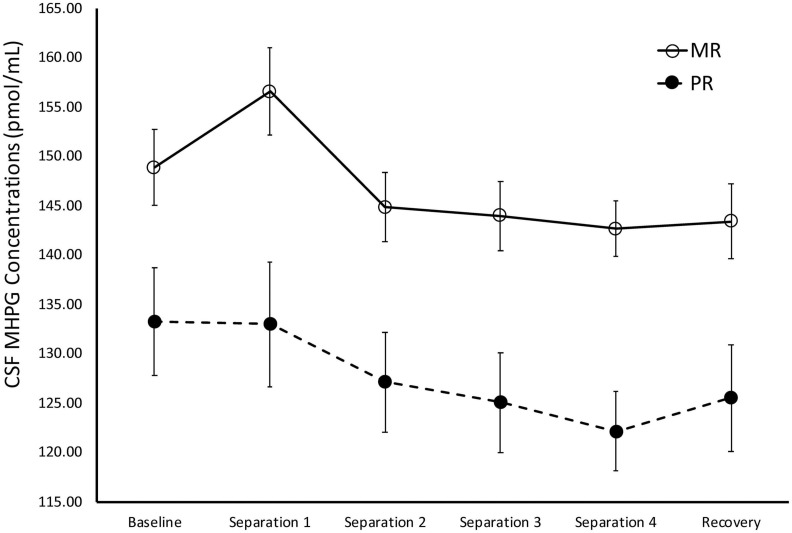
Rearing effects on baseline and stress-induced CSF MHPG concentrations. Depicts the relationship between rearing condition and CSF MHPG concentrations during *Baseline*, *Separations 1–4*, and *Recovery*. There was a significant main effect of time on CSF MHPG concentrations [*F*(1,141) = 10.21, *p* = 0.002], with CSF MHPG concentrations higher during *Baseline* and *Separations 1* and *2*, when compared to *Separations 3* and *4* or *Recovery*. There was also a significant main effect of rearing [*F*(1,141) = 14.15, *p* < 0.0001], with MR subjects exhibiting higher CSF MHPG concentrations at *Baseline*, *Separations 1–4*, and Recovery, when compared to PR subjects. MR subjects are represented by white circles and solid lines, PR subjects are represented by black circles and dashed lines.

There was a significant main effect of time on cisternal CSF MHPG concentrations [*F*(1,141) = 10.21, *p* = 0.002]. Further analyses showed that CSF MHPG concentrations obtained during *Baseline* and *Separations 1 and 2* were significantly higher than during the subsequent separations or the *Recovery* period (*p* < 0.05). There was also a significant main effect of rearing [*F*(1,141) = 14.15, *p* < 0.0001], with MR subjects exhibiting higher overall cisternal CSF MHPG concentrations (*M* = 146.74 ± 2.90), when compared to the PR subjects (*M* = 127.72 ± 4.14). MR subjects exhibited higher CSF MHPG concentrations at *Baseline (p* = 0.02), *Separation 1* (*p* = 0.003), *Separation 2* (*p* = 0.005), *Separation 3* (*p* = 0.003), *Separation 4* (*p* < 0.0001), and *Recovery* (*p* = 0.008), when compared to PR subjects. See Figure 3.

### Dopamine Metabolite (HVA)

**FIGURE 4:**
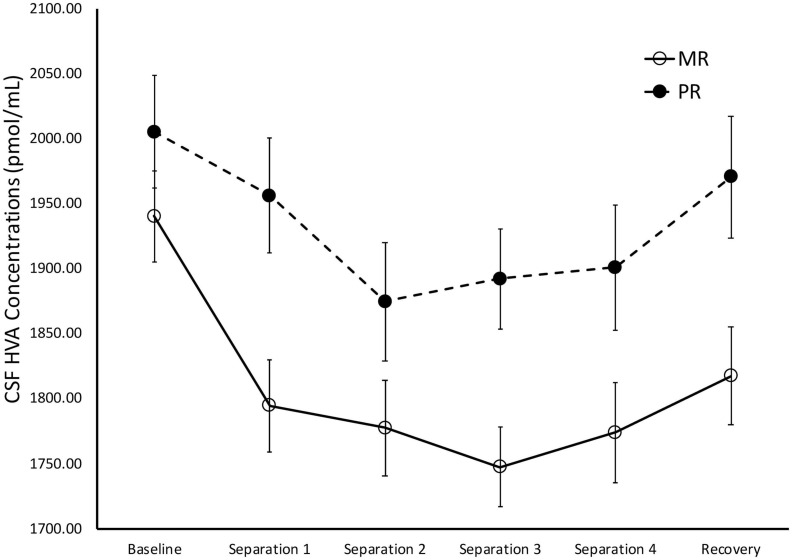
Rearing effects on baseline and stress-induced CSF HVA concentrations. Depicts the relationship between rearing condition and CSF HVA concentrations during *Baseline*, *Separations 1–4*, and *Recovery*. There was a significant main effect of time on CSF HVA concentrations [*F*(1,175) = 5.85, *p* = 0.02], with CSF HVA concentrations higher at *Baseline*, when compared to *Separations 1–4* or *Recovery*. There was also a significant main effect of rearing [*F*(1,175) = 8.36, *p* = 0.004], with MR subjects exhibiting lower CSF HVA concentrations at *Separations 1–4* and Recovery, when compared to PR subjects. MR subjects are represented by white circles and solid lines, PR subjects are represented by black circles and dashed lines.

There was a significant main effect of time on cisternal CSF HVA concentrations [*F*(1,175) = 5.85, *p* = 0.02)], with *Baseline* CSF HVA concentrations significantly higher than concentrations obtained during *Separations 1–4* or the *Recovery* period (*p* < 0.0001). There was also a significant main effect of rearing [*F*(1,175) = 8.36, *p* = 0.004], with MR subjects exhibiting lower overall cisternal CSF HVA concentrations (*M* = 1808.74 ± 27.92), when compared to PR subjects (*M* = 1933.47 ± 34.84). MR subjects exhibited lower CSF HVA concentrations at *Separation 1* (*p* = 0.005), *Separation 2* (*p* = 0.03), *Separation 3* (*p* = 0.003), *Separation 4* (*p* = 0.04), and *Recovery* (*p* = 0.01), when compared to PR subjects. MR and PR subjects’ CSF HVA concentrations did not differ at *Baseline (p* = 28). See Figure 4.

### 5-HTT Genotype and Serotonin Metabolite (5-HIAA)

**FIGURE 5:**
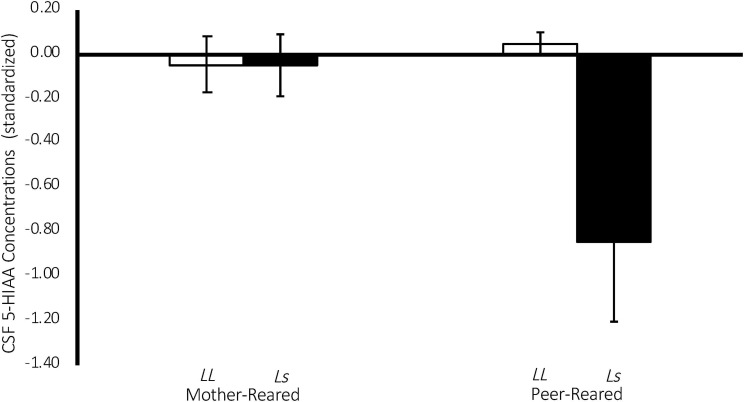
Interaction between 5-HTT genotype and rearing condition on CSF 5-HIAA Concentrations. There was a significant effect of 5-HTT genotype on CSF 5-HIAA concentrations [*F*(1,134) = 4.43, *p* = 0.04], with homozygous subjects exhibiting higher CSF 5-HIAA concentrations on average, when compared to subjects with an *s* allele. There was also a significant two-way rearing-by-5-HTT-genotype interaction on CSF 5-HIAA concentrations [*F*(1,134) = 4.29, *p* = 0.04], with PR subjects with an *s* allele exhibiting lower CSF 5-HIAA concentrations on average, when compared to MR subjects with an *s* allele, or to homozygous MR or PR subjects. MR subjects are represented in white, PR subjects are represented in black.

There was a significant effect of 5-HTT genotype on CSF 5-HIAA concentrations [*F*(1,134) = 4.43, *p* = 0.04], with homozygous subjects exhibiting higher CSF 5-HIAA concentrations on average (*M* = 427.10 ± 5.91), when compared to subjects with an *s* allele (*M* = 396.296 ± 9.682). Replicating an earlier study (Bennett et al., 2002), there was also a significant two-way rearing-by-5-HTT-genotype interaction on CSF 5-HIAA concentrations [*F*(1,134) = 4.29, *p* = 0.04], with PR subjects with an *s* allele exhibiting lower CSF 5-HIAA concentrations on average (*M* = 362.12 ± 16.43), when compared to MR subjects with an *s* allele (*M* = 430.47 ± 10.25), or to homozygous MR (*M* = 430.66 ± 7.73) or PR subjects (*M* = 423.54 ± 8.94). See Figure 5.

## Discussion

In line with the hypotheses, the results showed that early rearing experiences play a role in baseline monoamine metabolite concentrations and that monoamine metabolite concentrations are modulated by early rearing experiences during stress-inducing social separations and during a recovery period. This paper also replicates, for the first time the seminal findings of a gene-by-environment interaction effect on central serotonin metabolite levels, with PR subjects with the *s* allele exhibiting lower CSF 5-HIAA concentrations, when compared to homozygous PR subjects or to MR subjects with either genotype (Bennett et al., 2002).

MR subjects exhibited significantly higher CSF 5-HIAA concentrations before the stress-inducing separations, when compared to the PR subjects, consistent with a large number of earlier studies showing that baseline CSF 5-HIAA concentrations are modulated by early rearing experiences (Higley et al., 1992, 1996; Clarke et al., 1996; Shannon et al., 2005; Spinelli et al., 2010). However, during the stress-inducing social separations, the rearing effect on CSF 5-HIAA reversed, with the CSF 5-HIAA concentrations of MR subjects falling below that of the PR subjects, suggesting that the effects of early rearing experiences on the serotonin system are affected differentially by stress (see Figure 2). One potential interpretation of this finding is that, by nature of their early experiences, PR subjects develop a serotonin system that is less sensitive to changing situations, while MR subjects’ serotonin systems are more sensitive to stress-inducing conditions. As a percentage of baseline, the MR controls showed a much greater decline in cisternal CSF 5-HIAA concentrations across repeated separations than did the PR subjects (MR: 16% decline in cisternal CSF 5-HIAA levels; PR: 7% decline in cisternal CSF 5-HIAA levels). Unlike the PR subjects, the MR subjects showed a significant increase in CSF 5-HIAA concentrations during *Recovery*, although they did not completely return to *Baseline* concentrations. Future studies should extend the cisternal CSF sampling to investigate whether the MR subjects returned to their *Baseline* levels if given enough time. Other work from investigating the relationship between early rearing conditions and later life behavior follows a similar pattern: PR subjects show significantly greater alcohol intake, when compared to MR subjects during baseline, but the MR subjects increase their consumption to that of the PR subjects during a social separation stressor, nearly returning to baseline after being reunited with their the cage-mates (Higley et al., 1991a), a compelling similarity, given the relationship between low central serotonin and alcohol consumption in human and non-human primates (Higley et al., 1991a, 1996; LeMarquand et al., 1994).

These results suggest that a mother’s influence on her infant’s serotonin system not only assures normative levels of serotonin activity, but that, developmentally, mother’s maintain an infant’s responsiveness and sensitivity to environmental stimuli, particularly to stress. Absent the appropriate maternal input during this sensitive period, PR subjects exhibit lower serotonin activity at *Baseline* and less sensitivity and responsiveness during to stress, as evidenced by a reduced percent change in serotonin metabolite concentrations from *Baseline to Separations 1–4*. While speculative, it is possible that this may be related to the chronic stress that the PR subjects experience. Perhaps as a result of this, PR subjects showed relatively little change in CSF 5-HIAA concentrations when undergoing social separation procedures, while MR subjects, which, with exception to the social separation, live in the constant presence of their mother, exhibit drastic changes in CSF 5-HIAA concentrations. Given that mothers may help maintain their infant’s arousal during times of stress, it may be more stressful for MR subjects to undergo separation from their mothers, when compared to PR subjects’ separation from their peer groups. While it is beyond the scope of this study to mechanistically explain the relatively greater stress-induced change in CSF 5-HIAA concentrations in the MR subjects when compared to the PR subjects, Clarke et al. (1996) showed that, while MR subjects tend to exhibit stable, trait-like positive correlations in monoamine metabolite concentrations across development, PR subjects exhibit no such correlations. In another study, Kraemer et al. (1989) also showed that the three monoamine metabolites are intercorrelated across development in the MR subjects, but not in subjects reared without their mothers, arguing that the lack of a sensitive mother that reliably provides a secure base to reduce arousal, the hallmark of the attachment figures o individuals with a secure mother-infant attachment, is likely the source of this discrepancy.

During *Baseline*, stress-inducing *Separations*, and *Recovery* conditions, the maternally-deprived PR subjects exhibited significantly lower CSF MHPG concentrations, when compared to MR subjects (see Figure 3). These findings are in contrast to earlier work showing that early childhood maltreatment and trauma are associated with elevated norepinephrine concentrations (de Bellis et al., 1999; Otte et al., 2005), and illustrate the point discussed earlier that not all stressors are the same in their effect. Similarly, the PR subjects exhibited significantly higher CSF HVA concentrations during the stress-inducing *Separations* and *Recovery* conditions, when compared to MR subjects (see Figure 4). These findings are corroborated by earlier work showing that, when compared to children raised in homes where parents are sensitive to their infants’ emotional needs, abused and neglected children exhibit higher urinary dopamine concentrations (de Bellis et al., 1994, 1999; Roy, 2002; Egerton et al., 2016), as do children who experienced poor early maternal care, as measured by dopamine binding in PET assessment (Pruessner et al., 2004). These findings suggest that the effect of maternal absence leads to an atypical stress-response and potentially, an attenuated reward system. Using independent evolutionary theoretical models, Belsky (2002) and Ein-Dor et al. (2010) suggest that aberrations from typical development are not necessarily abnormal, but instead may be adaptations to the demands of the environment. In the case of PR subjects, one interpretation of their deviations from MR subjects in response to the stress of changing situations, is that the maternal absence they experienced early in life may have promoted adaptations to stressful situations, potentially leading to increased vigilance. These findings may also explain why PR subjects show reduced motivation and responses to rewarding stimuli (Paul et al., 2000; Nelson et al., 2009).

These findings suggest that a mother’s influence is instrumental in maintaining the interplay and dynamics between and within the monoamine systems. Regardless of the mechanism, overall, it is clear that maternal absence during this formative time has profound effects on the development of the monoamine systems, consistent with the theory that normative developmental processes are critically dependent on the right input at the right time, as proposed by Greenough et al. (1987). In this case, the right input is from mother, and her importance is punctuated by the differences between the MR and PR subjects in the response of the monoamine systems to stress. To paraphrase the now-classic words of Hebb (2005): in the absence of the right input (sensitive maternal response to arousal), at the right time, the systems failed to fire together, and, thus, failed to wire together.

Replicating earlier work (Bennett et al., 2002), there was a significant 5-HTT-genotype-by-rearing-condition interaction on CSF 5-HIAA concentrations, such that PR subjects with an *s* allele exhibited lower CSF 5-HIAA concentrations on average, when compared to MR subjects with an *s* allele, or to homozygous MR or PR subjects (see Figure 5). This finding evinces the long-term consequences of early rearing experiences on the serotonin system and that those consequences are made more or less extreme by experiential variation early in life. It is argued elsewhere that heritable influences on a variety of behaviors and developmental and psychopathological outcomes vary considerably across studies, indicating that genotypic effects are often dependent on the environment in which they are measured (Espinel and Higley, 2013). This replication punctuates this point, showing that CSF 5-HIAA concentrations during *Baseline* are lower in subjects with the *s* allele, but only if they are reared in the absence of mothers (PR).

While some studies with relatively small sample sizes indicate that the impact of early experience on the development of the monoamine systems persist beyond infancy (Higley et al., 1992, 1996), this study only included infant subjects. To investigate the long-term impact of early experience on the monoamine systems in later development, a longitudinal study including adult timepoints is of interest and would be an important contribution to the understanding of the persistence of the effects of early experience on monoamine functioning. Furthermore, it would be interesting to study the long-term predictive value of early experience-mediated monoamine functioning on behavioral and psychopathological outcomes.

Taken together, these findings indicate the importance of a sensitive caregiver early in life for normative neurodevelopment. These findings also suggest that stressful life events may elicit important differences in the monoamine metabolite systems, highlighting the importance of a caregiver that is sensitive to its infant’s emotional needs, which is the experience that the infant’s “experience-expectant” brain expects for normative development to occur. These findings are strengthened by the use of a rhesus monkey model, where the early rearing environment is randomly assigned and other extraneous variables can be closely controlled. Overall, these findings are an important step in understanding the impact of a caregiver that responds sensitively to its infant’s psychological needs early in life, leading to normative overall neurobiological development.

## Data Availability Statement

The raw data supporting the conclusions of this article will be made available by the authors, without undue reservation.

## Ethics Statement

The animal study was reviewed and approved by the NIH Animal Care and Use Committee.

## Author Contributions

EW and JDH were responsible for study concept and design. MS, SL, CB, SS, and JDH contributed to the acquisition of the data. EW, NG, AS, and JDH assisted with analyses and interpretation of the findings. EW and JDH drafted the initial manuscript. EW, NG, JH, AS, MS, SL, CB, SS, and JDH critically reviewed content and approved the final version of the manuscript for publication. All authors contributed to the article and approved the submitted version.

## Conflict of Interest

The authors declare that the research was conducted in the absence of any commercial or financial relationships that could be construed as a potential conflict of interest.

## Supplementary Material

Supplementary Figure 1 Representative Gel for 5-HTT Genotyping. Representative gel showing amplicons generated by PCR for the length variant in the regulatory region for the 5-HTT: going from left to right are the size standards and genotypes (*ss, Ls, LL, LL, ss, LL, Ls, LL, and LL*).
