# Supplementary figures and images for: Early Rearing Conditions Affect Monoamine Metabolite Levels During Baseline and Periods of Social Separation Stress: A Non-human Primate Model (Macaca mulatta)
Source: Front Hum Neurosci. 2021 Apr 9;15:624676. doi: 10.3389/fnhum.2021.624676 (PMC8062724; doi:10.3389/fnhum.2021.624676)

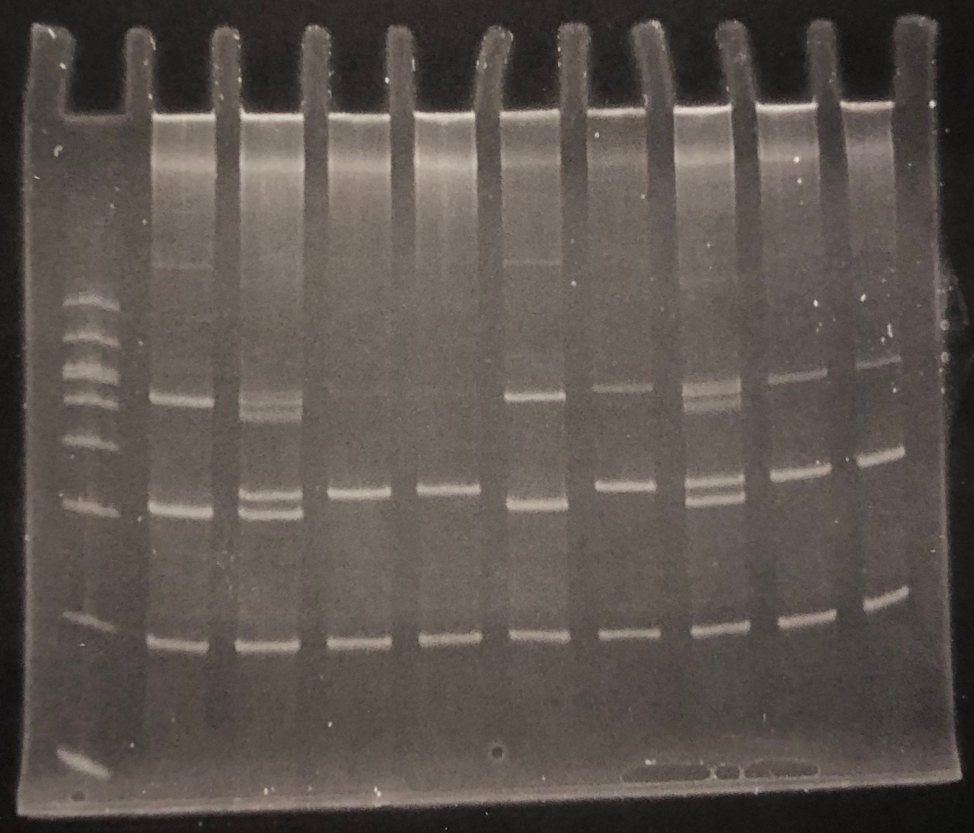

Supplement: Supplementary Figure 1 — Representative Gel for 5-HTT Genotyping. Representative gel showing amplicons generated by PCR for the length variant in the regulatory region for the 5-HTT: going from left to right are the size standards and genotypes (ss, Ls, LL, LL, ss, LL, Ls, LL, and LL). [file Image_1.PNG]
